# FOXE1 polyalanine tract length screening by MLPA in idiopathic premature ovarian failure

**DOI:** 10.1186/1477-7827-9-158

**Published:** 2011-12-16

**Authors:** Chun-rong Qin, Ji-long Yao, Wen-jie Zhu, Wei-qing Wu, Jian-sheng Xie

**Affiliations:** 1 Center for Reproductive Medicine, the Affiliated Shenzhen City Maternity and Child Healthcare Hospital of Southern Medical University, Shenzhen, Guangdong Province, PR China; 2 Department of Central Laboratory, the Affiliated Shenzhen City Maternity and Child Healthcare Hospital of Southern Medical University, Shenzhen, Guangdong Province, PR China

**Keywords:** premature ovarian failure, FOXE1, polyalanine tract, MLPA

## Abstract

**Background:**

FOXE1 is one of the candidate genes for genetic predisposition to premature ovarian failure (POF) and it contains an alanine tract. Our purpose is to assess the influence of length of the alanine tract of FOXE1 on genetic susceptibility to POF.

**Methods:**

The group studied consisted of 110 Chinese patients with idiopathic POF and 110 women from normal controls. The polyalanine tract and flanking sequence of FOXE1 was screened using the Multiple Ligation-dependent Probe Amplification (MLPA) technique and directly sequenced.

**Results:**

Three variants of FOXE1-polyalanine length, containing 12, 14, or 16 alanine residues, and 5 different genotypes were identified. There were significantly lower frequencies of the 14/14 genotypes in cases with POF (X2 = 119.73, P = 0.001), as compared with the controls. The incidence of 16/16 genotypes of FOXE1-polyalanine was significantly higher in patients with POF (X2 = 3.403, P = 0.001) in comparison to the controls. The FOXE1 14 alanine allele was significantly less common in the POF patient group (186/220) than the controls (216/220) (X2 = 25.923, P = 0.0001). The FOXE1 16 alanine allele was significantly more common in the POF patient group (28/220) than the controls (4/220) (X2 = 19.412, P = 0.0001).

**Conclusion:**

This finding provides evidence that polyalanine repeat expansions in FOXE1 may be responsible for the genetic aetiology of POF in Chinese women.

## Background

The diagnosis of premature ovarian failure (POF) is based on the finding of amenorrhea before the age of 40 years associated with follicle-stimulating hormone levels in the menopausal range. It is a common disease affecting approximately 1% of women <40 years, 1:10,000 women by age 20 years and 1:1,000 women by age 30 years [1]. POF is generally characterized by low levels of gonadal hormones and high levels of gonadotropins (LH and FSH). POF is a heterogeneous disorder determined by many pathogenic mechanisms [2]. The principal causes of POF are chromosomal, genetic, autoimmune, metabolic, infectious, and iatrogenic. A large proportion of cases remain without a known cause, and these are classified as idiopathic or karyotypically normal spontaneous premature failure [2].

In the last 2 decades, many studies using molecular biology technology aimed to discover a relationship between POF and genetic disorders [3-6]. POF has recently been associated with mutations in Forkhead L2 (FOXL2) gene, which is associated with blepharophimosis-ptosis-epicanthus inversus (BPES) syndrome [7]. BPES is a rare autosomal dominant disease with a prevalence of about 1 in 50,000. Clinically, BPES has been divided into two subsets depending on the association of ocular malformation with (type I) or without (type II) POF. Genetically, however, both types are caused by mutations in FOXL2, and a genotype-phenotype correlation has been described in some cases [8].

FOXL2 belongs to the large family of forkhead (FOX) transcription factors which encodes a transcription factor containing a forkhead domain for DNA-binding and a polyalanine domain of uncertain function. Members of this family are expressed in a wide range of tissues, are involved in a variety of developmental processes and are thought to play an important role in mediating transforming growth factor (TGF) superfamily signals by binding to members of the Smad family of proteins [9].

Following the discovery of FOXL2 polyalanine tract deletions in POF patients [10,11], it was important to study other forkhead genes with a polyalanine tract in order to determine whether changes in this region might also be associated with POF. FOXE1 consists of a single exon that codes for a forkhead domain, a polyalanine tract, and unique C-terminal residues. Initially, the polyalanine tract was reported to consist of 19 residues [12]. Subsequently, the length of the major alanine stretch was shown to be 14 residues [13]. Patients carrying homozygous mutations in FOXE1 present athyroidal hypothyroidism, spiky hair, choanal atresia, cleft palate and bifid epiglottis, known as Bamforth-Lazarus syndrome [14]. Polymorphism of the polyalanine tract of FOXE1 was reported for the first time by Macchia et al. with variable length from 12 to 17 alanines [13]. However, it is noteworthy that frequency of alleles is quite different among the different control groups, even among the same population [13,15]. A few studies have pointed to the potential role of FOXE1-polyalanine length polymorphism in determining the susceptibility to POF [16]. However, the evaluation of its length in chinese patients with POF has not been conducted so far. Hence, the objective of the present study was to assess the influence of the FOXE1-polyalanine length on susceptibility to POF in Chinese women, using the Multiple Ligation-dependent Probe Amplification (MLPA) technique, which is one of the best methods for detecting alterations in gene dosage [17].

## Methods

### Patient and control recruitment

One hundred and ten patients with idiopathic POF were recruited between January 2009 and July 2010 at the Affiliated Shenzhen City Maternity and Child Healthcare Hospital of Southern Medical University, Shenzhen, PR China. The study was approved by the University's Institutional Ethics Committee and informed consent was obtained from all participants. The diagnostic criteria for POF was as follows: at least 6 months of amenorrhoea before the age of 40, with at least two serum FSH concentrations of >40 IU/l. Controls (n = 110) were individuals under 40 with proven fertility, normal menstrual cycles, normal FSH levels and ovary morphology, with no history of subfertility treatment. Each patient and control were assessed clinically, with a complete medical and gynecological history, including the history of menses, age at menopause, LH and FSH levels (two times at one-month intervals), T_3_,T_4 _and TSH levels, and pelvic ultrasound. Patients with associated endocrinopathies, autoimmune disorders, iatrogenic agents, such as pelvic surgery, chemotherapy, and radiotherapy, and infections, were excluded. The controls were recruited from the health examination department of the Affiliated Shenzhen City Maternity and Child Healthcare Hospital of Southern Medical University, matched by sex and age. In the control group, tumor, endocrinopathies, autoimmune disease, and infections were excluded. Karyotyping with high-resolution GTG banding to check for chromosomal anomalies was performed in all patients and controls. Those with abnormalities were excluded from the study.

### DNA extraction and karyotyping

A 5 ml aliquot of peripheral blood was collected in EDTA vacutainers for genomic DNA isolation, and another 5 ml of peripheral blood was collected in heparin vacutainers for chromosomal analysis. Genomic DNA was extracted from lymphocytes using standard proteinase K/chloroform extraction methods [18]. Chromosomal analysis was performed on phytohaemagglutinin (PHA)-stimulated peripheral lymphocyte cultures using standard conventional cytogenetic methods.

### PCR

The FOXE1 (Swiss-Prot: O00358) coding sequence, flanking the polyalanine tract, was amplified using standard polymerase chain reaction (PCR) conditions. The following primers, designed to border polyalanine tract, were used : forward (F): 5´-CTTCAAGCGCTCGGACTCTC-3´ and reverse (R): 5´-ACGCCGCGGGGTAGTAGACTG-3´. The position of the primers was c.444-c.749.The amplicons (194 bp to 212 bp,12-18 alanines) were purified. After 4 min of initial denaturation at 95°C, 40 cycles of PCR were performed , followed by 7 min of final extension at 72°C. Each cycle included: denaturation 30 s at 95°C, annealing 30 s at 64°C, and elongation 30 s at 72°C. PCR was performed in 25 μl volume of reaction mixture, with the following reagents concentrations: 2.5 μl of 10**×**PCR buffer with Mg, 5 μl of GC-rich solution, 2.5 μl of dNTP at a final concentration of 200 μM, 0.2 μl of Fast Start Taq Polymerase (Roche Diagnostics, Mannheim, Germany), 2 μl of DNA (150 ng), 1 μl of primers (F+R, final concentration of 1 μM) and 11.8 μl of nuclease-free water.

### MLPA Analysis

All the MLPA reagents come from MRC-Holland (Amsterdam the Netherlands). The hybridization and ligation of probes were performed in Biosystems 2720 thermal cycler according to the standard MLPA protocol. pCYF (5´CACGACGTTGTAAAACGACCTTCAAGCGCTCGGACCTCTC-3´) and pCYR (5´-ACGCCGCGGGGTAGTAGACTG-3´) were used as universal PCR primers in the ligated probes amplification. The length of amplicons obtained ranged from 213 bp (corresponding to 12-alanine polyalanine) to 231 bp (corresponding to 18-alanine polyalanine). 250 ng DNA of each patient was denatured for 5 min at 98**°C **and hybridized overnight at 60**°C **with the SALSA probe mix pCYF and pCYR. The hybridization products were subsequently treated with a ligase enzyme for 15 min at 54**°C**. The ligation reaction was stopped by raising the temperature for 5 min at 98**°C**. Finally, PCR amplification was carried out with the specific SALSA FAM primers using the ligation product as templates. PCR products were checked on a 2% agarose gel and subsequently resolved by capillary electrophoresis on an ABI PRISM 3130. The data were analyzed by the Gene Marker version 1.6 software.

All PCR products were obtained using the above-mentioned primers, amplifying the polyalanine tract and flanking regions of the gene. Samples were sequenced using BigDye Terminator Cycle Sequencing Kit 3.1(Applied Biosystems, USA) with the above primers, and run on a 3730 × l ABI DNA Analyzer (Applied Biosystems). The sequencing results were analyzed using Chromas (version 2.3) and compared with reference sequences in the National Center for Biotechnology Information (NCBI) database.

### Statistical analysis

Chi-square test was performed using SPSS 13.0 Software (SPSS Inc., Chicago, IL). We used two-sided Fisher's exact test to compare Ala polymorphisms between POF and controls.

## Results

### Clinical characteristics of the population

One hundred and ten Chinese patients with idiopathic POF and 110 women from normal controls completed the study. Mean age was 31.58 ± 6.02 years (18-39) in the POF patients and 30.43 ± 4.02 years (20-39) in the controls. Most of our patients (105/110; 95.5%) presented with normal puberty and secondary amenorrhea, and only 5 (4.5%) displayed primary amenorrhea.

### Sequence variants detected in FOXE1

Assessment of the FOXE1-polyalanine length was performed for the patients with POF and the control group. In the population studied, FOXE1-polyalanine was polymorphic in both groups. Three variants of FOXE1-polyalanine length, containing 12, 14, or 16 alanine residues, and 5 different genotypes were identified (Table 1). The most common genotypes were 14/14 homozygote, occurring with the frequency of 81.2 % in the POF group, while 96.4% in control subjects, respectively. There were significantly lower frequencies of the 14/14 genotypes in cases with POF (χ^2 ^= 119.73, P = 0.001), as compared with the controls. The homozygote for 16/16 residues was identified in 10.0% of the patients with POF, but was not found in control subjects. Compared with controls, there were significantly higher frequencies of the 16/16 genotypes in cases with POF (χ^2 ^= 3.403, P = 0.001). The heterozygote for 14/16 residues was identified in 2.7% of the patients with POF, but also in 3.6% of the control subjects. The rare 12/14 heterozygous FOXE1 variant as well as the 12/16 homozygous variant were only detected in POF subjects with a frequency of 2.7%, but was not found in control subjects.

**Table 1:** Percentage of identified FOXE1 genotypes frequency in patients with premature ovarian failure (POF) and a control group

Genotypes	POF n (%)	Controls n (%)	χ^2^	P
14/14	90(81.2)	106(96.4)	119.73	0.001^a^
16/16	11(10.0)	0(0)	3.403	0.001 ^a^
12/14	3(2.7)	0(0)	3.041	0.247
12/16	3(2.7)	0(0)	3.041	0.247
14/16	3(2.7)	4(3.6)	0.148	1.000

a P Value remains significant after Fisher's exact test with Bonferroni's correction for multiple testing: p value = 0.010.

Fisher's exact test was used to compare individual allele frequencies between POF and controls. The FOXE1 14 alanine allele was significantly less common in the POF patient group (186/220) than the controls (216/220) (χ^2 ^= 25.923, P = 0.0001). The FOXE1 16 alanine allele was significantly more common in the POF patient group (28/220) than the controls (4/220) (χ^2 ^= 19.412, P = 0.0001).

Bonferroni's adjustment to an overall significance level of 0.05 was used as the cutoff value (i.e., α = 0.05/n, where "n" is the number of tested). Finally, the use of arbitrary a priori P value cutoffs in conjunction with Bonferroni's correction proved useful in the analysis of microarray data [19], so data in this study was also examined at two P values, 0.010 for genotypes frequency analysis and 0.016 for allele frequency analysis. After Bonferroni's adjustment for multiple statistical analyses, the above findings remain significant. The total distribution of different alleles is summarized in Table 2.

**Table 2:** Percentage of identified FOXE1 allele frequency in patients with premature ovarian failure (POF) and a control group

Allele	POF n (%)	Controls n (%)	χ^2^	P
12	6(2.7)	0(0)	6.083	0.0300
14	186(84.5)	216(98.2)	25.923	0.0001 ^a^
16	28(12.7)	4(1.8)	19.412	0.0001 ^a^

a P Value remains significant after Fisher's exact test with Bonferroni's correction for multiple testing: p value = 0.016.

**Figure 1:**
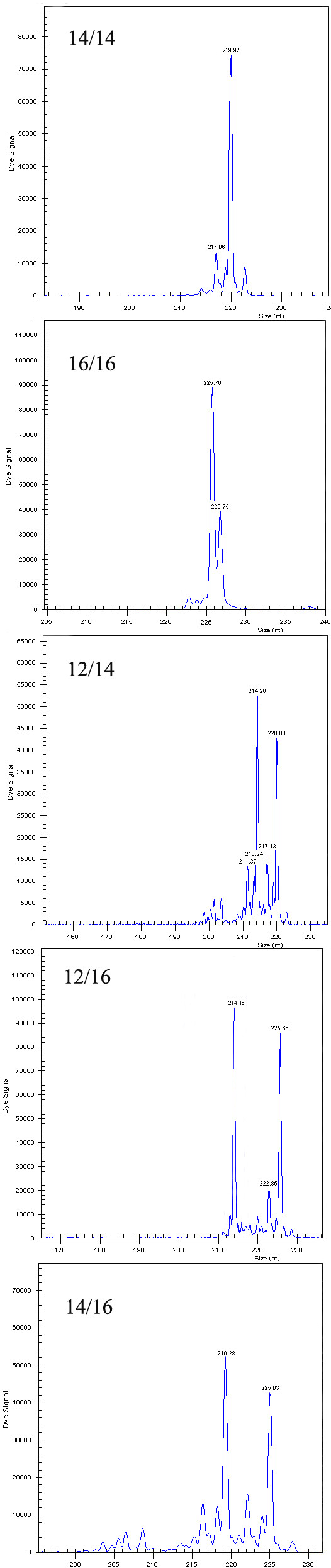
**Analysis of FOXE1-polyalanine length with the use of Multiple Ligation-dependent Probe Amplification (MLPA) technique**. Pherograms displaying the MLPA output of the five different FOXE1 genotypes. In the Y-axis are depicted the intensity signals (peak heights) for probe that are depicted in the X-axis according to their length (probe size).

The preferred screening approach was to use MLPA to size the different alleles. Pherograms displaying the MLPA output of the five different FOXE1 genotypes were shown in Figure 1.

**Figure 2:**
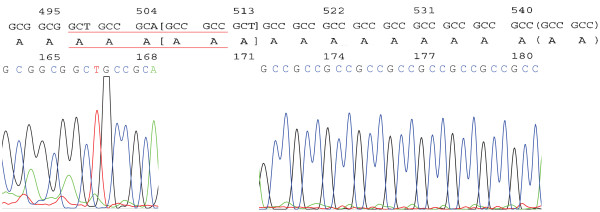
**Nucleotide sequence and its corresponding amino acid sequence for the FOXE1 gene around the polyalanine tract**. 496-510del (AAAAA166-170del) is shown by underlining, 505-513del (AAA169-171del) is shown by [ ], and 541-546del (AA181-182del) is shown by ().

These reduced polyalanine residues of 496-510del(AAAAA166-170del), 505-513del(AAA169-171del), and 541-546del(AA181-182del) were detected in the patients with POF and the control group. Three types of missing alanine residues were shown in Figure 2.

## Discussion

Alanine-tract expansions in transcription factors have been implicated as a cause of some human diseases. The polyalanine tract length of FOXE1 is of interest as FOXL2, a gene commonly mutated in BPES and more recently found to be altered in patients with isolated POF, contains a highly conserved polyalanine tract [8,11]. FOXE1 is located at 9q22 and the single exon gene encodes a forkhead domain, a polyalanine tract, and unique C-terminal residues. It is thought to be involved in thyroid morphogenesis. Mutations in FOXE1 cause Bamforth-Lazarus Syndrome. FOXE1 polyalanine tract length has not been specifically correlated with these thyroid dysfunctions [20]. However, it is possible that certain alleles, or certain alleles in combination, are linked to a higher risk of thyroid complications. It was noted that between 10 and 20% of women with POF have an autoimmune disease, most commonly hypothyroidism [21]. FOXE1 is therefore an appropriate gene to investigate, in order to identify more genetic causes of POF. The length of FOXE1 -polyalanine reported in this study varied from 12 to 16 alanines, compared to previous reports [13,16,22,23] where a total number of alanine coding triplets ranged from 11 to 19. The observed inconsistency could be at least partially explained with ethnic differences of polyalanine tract variations.

To date, polyalanine tract expansions in transcription factors have been reported as a molecular factor of numerous diseases and elongation of the tract was connected with higher morbidity, a more severe clinical picture and poorer prognosis [24]. On the other hand, polyalanine tract contractions have not been involved with certainty in the etiopathogenesis of human diseases [25].A computer program that predicts protein secondary structure [26] indicated that the polyalanine tract of FOXE1 constitutes an α-helical region C-terminal to the forkhead domain. In several transcription factors which repress the transcription of target genes, alanine-rich regions, which form α-helical regions and are responsible for the transcriptional repression, were identified [27,28]. Alanine expansion may lead to disturbances in protein conformation and potentially affect the process of transcription regulation by impairing specific binding to DNA.

In this study of 110 Chinese population with idiopathic POF, we detected no mutations of the FOXE1 gene other than an already known silent single nucleotide polymorphism and polymorphic expansions or contractions of the alanine-encoding nucleotide triplets that lead to variations of the length of alanine tracts within the polyalanine domain of the FOXE1 gene product. Recently, in the study on a Poland population by Szczepanek et al [29], the incidence of longer variants (≥16 codons) of FOXE1-polyalanine was significantly higher in patients with the familial form of thyroid hemiagenesis(TH) in comparison to those with sporadic TH. Our finding of polymorphisms is in accordance with the studies from Watkins et al [16]. We found that the homozygous Ala14 polymorphism (Ala14/14) was less frequent in the POF group than in the controls. In contrast, significantly more POF patients than controls harbored the Ala16 polymorphism(Ala16/16). Indeed, patients affected by POF present a significantly lower proportion of the 14/14 genotype compared to controls (81.2% vs. 96.4%). Thus, POF is associated with the more common variant (allele 16) suggesting that the more common variant (allele 16) may increases the risk of developing POF. Our results suggest that the length of the alanine stretch within FOXE1 modulates genetic susceptibility to POF. Interestingly, we have discovered three different reduced polyalanine residues which make up the 12-16 alanine FOXE1 alleles leading us to speculate that different mechanisms have operated to bring about these contractions and expansions. The lack of a variation in the Chinese population of the alleles (17, or 19) is interesting, as we assume that this is from a relatively homogenous population, and is similar to what is seen in the Japanese population [20], but not in some of the New Zealand populations [16] studied to date. These difference might reflect ethnicity-related deviations.

Previous studies employed direct sequencing [29] and DHPLC [16] for analysis of FOXE1-polyalanine length polymorphism, while in our study an analysis of DNA fragment length was performed with MLPA. Major drawbacks of the DHPLC method are chemical waste, high maintenance cost, the need for post-PCR manipulations, and low throughput [30]. Unlike DHPLC, MLPA is a recently developed semi-quantitative method that aims to detect copy number alterations at the genomic level (gains or loses) in a test DNA with respect to a control. Due to its low cost, reliability and ease of implementation it has become very popular both as a research and a diagnostic tool [31]. This appeared to be a useful method for the evaluation of polyalanine tract expansions as its consistency with direct bilateral sequencing results was confirmed for selected samples. Moreover, interpretation of sequences obtained in direct sequencing from heterozygous patients is a difficult and mistake-prone method, while MLPA is unequivocal and easy (Figure 1). As until now this technique have not been reported to screen the FOXE1 gene, we applied the usefulness of MLPA as a method to detect sequence alterations in the polyalanine tract of FOXE1.

## Conclusions

To sum up, the present study confirmed a high heterogeneity of the number of alanine residues in FOXE1 in the population studied. The heterogeneity was identified even among the control subjects, therefore indicating polymorphic variability and a rather weak impact on phenotype. However, A significant difference in genotype and allele frequencies were noted between the two groups, which can be attributed to the increased frequency of 16/16 homozygotes in the POF group. There is no evidence for an increase in heterozygous carriers of the 16 allele suggesting this affects susceptibility in a recessive manner. The results obtained suggest that alteration from the most frequently occurring 14 alanine allele to the 16 allele increases the risk of developing POF, therefore FOXE1-polyalanine tract expansion may contribute to the molecular background of POF. Still, the number of patients studied sets limits in the interpretation of the results obtained, the same as does the fact that no molecular study has been performed to support the functional significance of FOXE1-polyalanine length polymorphism. More research is required to confirm such findings.

## Competing interests

The authors declare that they have no competing interests.

## Authors' contributions

CRQ contributed to conception, design, and initiation of the study. JLY performed the clinical procedures. WQW made substantial contributions to the analysis of samples and data. WJZ, and JSX contributed to the interpretation of the data and the preparation of the manuscript. All authors read and approved the final manuscript.
